# Examining the Distribution and Impact of Single-Nucleotide Polymorphisms in the Capsular Locus of Streptococcus pneumoniae Serotype 19A

**DOI:** 10.1128/IAI.00246-21

**Published:** 2021-10-15

**Authors:** D. W. Arends, W. R. Miellet, J. D. Langereis, T. H. A. Ederveen, C. E. van der Gaast–de Jongh, M. van Scherpenzeel, M. J. Knol, N. M. van Sorge, D. J. Lefeber, K. Trzciński, E. A. M. Sanders, H. C. Dorfmueller, H. J. Bootsma, M. I. de Jonge

**Affiliations:** a Laboratory of Medical Immunology, Radboud Center for Infectious Diseases, Radboud Institute for Molecular Sciences, Radboud University Medical Center, Nijmegen, The Netherlands; b National Institute for Public Health and the Environment, Bilthoven, The Netherlands; c Center for Molecular and Biomolecular Informatics, Radboud Institute for Molecular Life Sciences, Radboud University Medical Center, Nijmegen, The Netherlands; d GlycoMScan, Oss, The Netherlands; e Translational Metabolic Laboratory, Department of Neurology, Radboud University Medical Center, Nijmegen, The Netherlands; f Department of Medical Microbiology and Infection Prevention, Netherlands Reference Laboratory for Bacterial Meningitis, Amsterdam Institute for Infection and Immunity, Amsterdam University Medical Center, University of Amsterdam, Amsterdam, The Netherlands; g Department of Paediatric Immunology and Infectious Diseases, Wilhelmina Children’s Hospital, University Medical Center Utrecht, Utrecht, The Netherlands; h Division of Molecular Microbiology, School of Life Sciences, University of Dundee, Dundee, United Kingdom; University of Illinois at Chicago

**Keywords:** Dutch cohort, *Streptococcus pneumoniae*, capsule shedding, polysaccharide capsule, serotype 19A, whole-genome sequencing

## Abstract

Streptococcus pneumoniae serotype 19A prevalence has increased after the implementation of the PCV7 and PCV10 vaccines. In this study, we have provided, with high accuracy, the genetic diversity of the 19A serotype in a cohort of Dutch invasive pneumococcal disease patients and asymptomatic carriers obtained in the period from 2004 to 2016. The whole genomes of the 338 pneumococcal isolates in this cohort were sequenced and their capsule (*cps*) loci compared to examine their diversity and determine the impact on the production of capsular polysaccharide (CPS) sugar precursors and CPS shedding. We discovered 79 types with a unique *cps* locus sequence. Most variation was observed in the *rmlB* and *rmlD* genes of the TDP-Rha synthesis pathway and in the *wzg* gene, which is of unknown function. Interestingly, gene variation in the *cps* locus was conserved in multiple alleles. Using RmlB and RmlD protein models, we predict that enzymatic function is not affected by the single-nucleotide polymorphisms as identified. To determine if RmlB and RmlD function was affected, we analyzed nucleotide sugar levels using ultrahigh-performance liquid chromatography–mass spectrometry (UHPLC-MS). CPS precursors differed between 19A *cps* locus subtypes, including TDP-Rha, but no clear correlation was observed. Also, significant differences in multiple nucleotide sugar levels were observed between phylogenetically branched groups. Because of indications of a role for Wzg in capsule shedding, we analyzed if this was affected. No clear indication of a direct role in shedding was found. We thus describe genotypic variety in *rmlB*, *rmlD*, and *wzg* in serotype 19A in the Netherlands, for which we have not discovered an associated phenotype.

## INTRODUCTION

Streptococcus pneumoniae is a common resident of the human upper respiratory tract. It can disseminate into the lungs, causing pneumonia, and invade the bloodstream, leading to sepsis and meningitis. Invasive infections give rise to high morbidity and mortality rates worldwide, especially in young children, the elderly, and immunocompromised individuals. Pneumococcal disease often occurs as a coinfection or secondary infection, especially in influenza or influenza-like illness. It is also a common cause of otitis media in children (1).

S. pneumoniae produces capsular polysaccharides (CPS), which are important virulence factors. The CPS was shown to protect the bacterium against complement-mediated opsonophagocytosis and multiple other antibacterial pathways by shielding its immunogenic surface proteins from binding by host factors, such as complement factors or antibodies (2–5). Virulence is affected by capsule thickness, charge, and chemical properties (1, 6, 7). The negatively charged CPS is thought to promote colonization of the upper respiratory tract by repelling host mucopolysaccharides, which reduces mucosal clearance (8). S. pneumoniae serotype is determined by the polysaccharide antigen, and to date, around 100 have been described (https://www.pneumogen.net/gps/serotypes.html). For almost all serotypes, genes encoding the formation of the capsule are located in the capsular gene locus, an operon regulated through a single promoter region. The *cps* locus is located between *dexB* and *aliA* (9). Because of its important role in virulence and its accessibility to the immune system, the CPS has been the main target for vaccine development.

Multiple S. pneumoniae vaccines have been developed, of which pneumococcal conjugate vaccines (PCVs) targeting the CPS of specific serotypes not only protect against disease but also interfere with transmission by prevention of colonization of the nasopharynx (10, 11). After implementation of the PCV7 vaccine, targeting S. pneumoniae serotypes 4, 6B, 9V, 14, 18C, 19F, and 23F, the prevalence of invasive pneumococcal disease (IPD) cases caused by nonvaccine types has increased across multiple sites (11), including in the Netherlands (12), as a consequence of serotype replacement and/or capsule switching. In the early 2010s, new conjugate vaccines were developed that covered additional serotypes, namely PCV10 (PCV7 serotypes plus 1, 5, and 7F) and PCV13 (PCV10 serotypes plus 3, 6A, and 19A). Most Western European countries have implemented the PCV13 vaccine, while the Netherlands has implemented and is still using PCV10. There is limited evidence for vaccine cross-protection, i.e., protection provided by antibodies raised against a specific serotype binding to the CPS of another serotype with a similar structure. Despite high structural similarity with 19F, limited protection against 19A infection was found after PCV10 vaccination (13–15). In other studies, a complete absence of cross-protection was observed (16, 17), and several studies were inconclusive (18, 19). In most countries that have implemented PCV10, 19A prevalence has been on the rise (11). In countries in Western Europe using PCV10, a marked decrease in PCV10-serotype IPD was reported; however, the proportion of IPD due to PCV13-unique serotypes remained high at 5 to 64%, predominantly due to serotypes 19A and 3. Although PCV13 implementation has diminished the prevalence of 19A in most of these countries, 19A is one of the most frequently found emerging serotypes, even in some countries that have implemented PCV13 (20). Also, several cases of 19A infections in hospitalized individuals vaccinated with PCV13 have been reported (21). Therefore, examination of the 19A serotype remains relevant, despite an increasing number of countries that are implementing PCV13.

**FIG 1:**
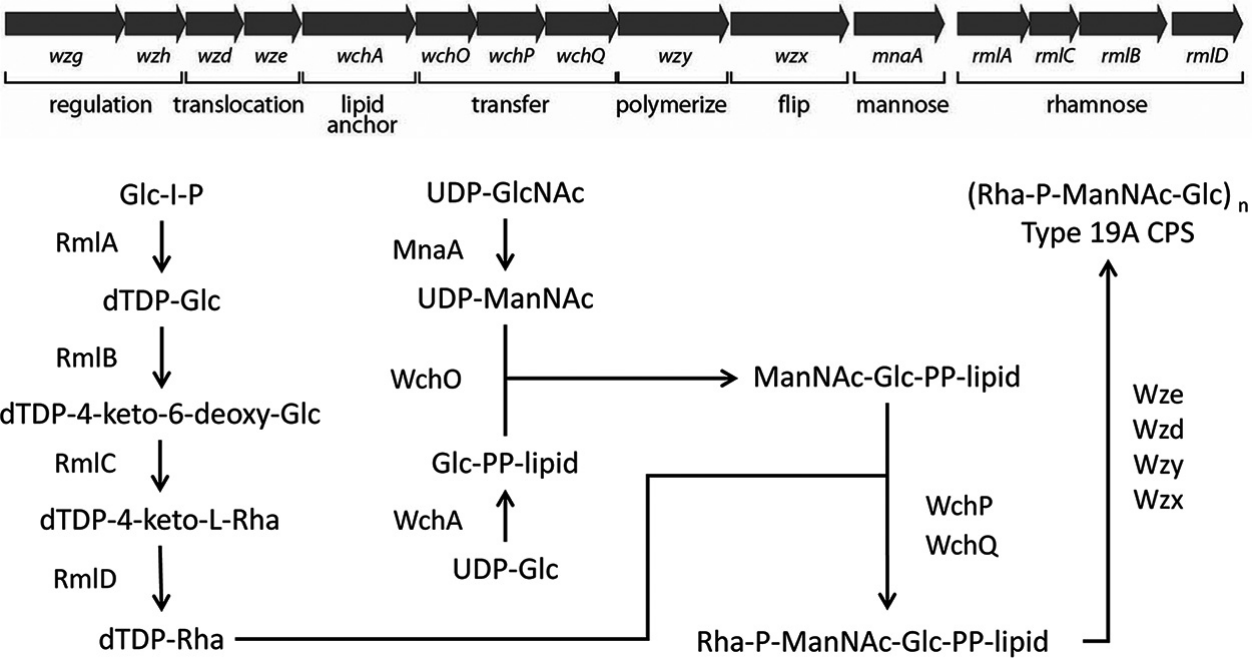
(Top) The capsule locus of S. pneumoniae serotype 19A and the function of its genes in the production of the capsular polysaccharide (CPS) capsule. (Bottom) Biosynthetic pathway of the serotype 19A capsular polysaccharide. Derived from Morona et al. (22).

Serotype 19A belongs to serotype group 19, which contains 19A, 19B, 19C and 19F. The capsules of 19F and 19A differ only in the link between glucose (Glc) and rhamnose (Rha): 1→2 or 1→3, respectively (22). However, the genes in the *cps* locus are relatively different (70 to 99% similarity) (22). Based on different alleles within the *cps* locus (Fig. 1), multiple 19A subtypes have been described (23). Generally, the highest sequence diversity is in the regulatory gene *wzg* and in the TDP-Rha biosynthesis genes *rmlC*, *rmlB*, and *rmlD* (23). In some subtypes, the *rmlD* gene has been flipped and is in a reverse position (3′–5′) in the locus (23). The *rml* genes are all required for TDP-Rha biosynthesis, which in turn is required for 19A polysaccharide capsule production. RmlA forms a tetramer and converts glucose-1-phosphate into TDP-glucose, which is oxidized and dehydrated by a RmlB dimer to form TDP-4-keto-6-deoxy-d-glucose. Dimeric RmlC in turn catalyzes a double epimerization reaction, after which RmlD monomers forms TDP-l-rhamnose (24). The end product of the biosynthetic pathway of the 19A CPS is a polymer of a repeat unit trisaccharide TDP-Rha-P-ManNAc-Glc (Fig. 1), which is connected to the cell wall peptidoglycan (22).

The function of *wzg* is unknown, making it difficult to speculate about the effect of variation within *wzg* sequence. Wzg is required for normal CPS levels. It was shown to possess enzymatic properties to anchor the CPS to the peptidoglycan cell wall (25, 26), which might be involved in capsule shedding, the release of CPS by the bacteria. This might function as a decoy for antibody binding, leading to immune evasion. Increased capsule shedding could also increase the likelihood of epithelial cell invasion and host-host transmission (26–28). The consequences of the observed genetic diversity in the *cps* locus of 19A are still unclear. In this study, we describe the genetic diversity of the 19A serotype in the Dutch population and our attempt to reveal its consequences and driving forces.

We subjected strains collected from the nasopharyngeal or oropharyngeal cavity in healthy individuals (carriage strains) and strains collected from clinical cases of S. pneumoniae infection (IPD strains) to whole-genome sequencing. Samples ranged across the period before and after implementation of the PCV7 and PCV10 vaccines. The *cps* locus sequences were compared to examine whether certain single-nucleotide polymorphisms (SNPs) and/or 19A subtypes were associated with carriage or disease and to characterize the diversity over time upon potential selective pressure by both PCV vaccines. Furthermore, nucleotide sugars, including TDP-Rha, were measured, and structural modeling on RmlB and D was performed in order to understand the impact of SNP accumulation in genes encoding these proteins. Additionally, we examined capsule shedding for the different Wzg proteins we observed.

## RESULTS

### Sequence variation in the *cps* locus and the identification of 19A subtypes.

Considerable DNA sequence variation within the 19A *cps* locus was reported previously (23, 29), referred to as capsular subtypes. Elberse et al. (23) described that after implementation of the PCV7 vaccine (in 2006), an apparent shift in the prevalence of 19A capsular subtypes in invasive pneumococcal disease (IPD) was observed in the Netherlands, from a majority of 19A-I to a majority of 19A-II (2004 and 2005 versus 2008 and 2009). A similar shift after PCV7 implementation in Switzerland was also observed by Brugger et al. (29). We investigated whether the post-PCV7 emergence of 19A-II continued using the same allele-specific screening PCR methodology. However, the distribution of capsular subtypes in recent years (2013 to 2016) seems to be returning to the distribution observed before implementation of PCV vaccination (see Fig. S1 in the supplemental material).

**FIG 2:**
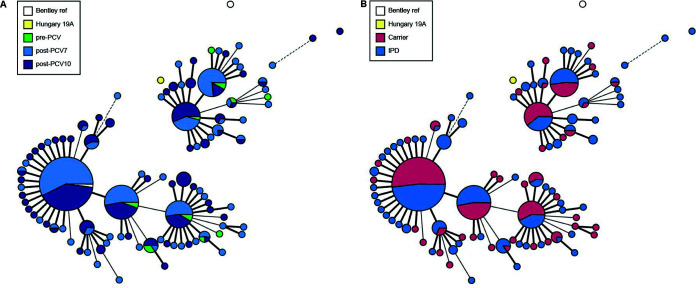
Minimum spanning tree of a multilocus sequence typing (MLST) analysis of the 19A serotype strains of our cohort (*n* = 338). MLST analysis was based on the sequence of the *cps* locus. Strains are grouped based on origin of samples by time period (A) or type of host (B).

For a more detailed view on the differences in 19A *cps* locus sequence, we subjected pneumococcal strains isolated from carriage (*n* = 148) and IPD isolates (*n* = 188, Pneumococcal Bacteraemia Collection Nijmegen [PBCN] cohort [30]) to whole-genome sequencing and extracted the complete *cps* locus sequence from the assemblies (Table 1). Initial comparison of the *cps* gene cluster sequences was performed by cpsMLST, a serotype-specific multilocus sequence typing (MLST)-like scheme based on *cps* genes. Among the 338 isolates from which complete *cps* sequences were available, 100 unique cpsMLST types were detected, providing higher resolution than the previously defined capsular subtypes identified by PCR (Fig. S2). The cpsMLST types correlated with PCR subtypes, but additional genetic variation within the subtype could be observed. No particular cpsMLST type could be linked to either isolation period (before and after PCV vaccination; Fig. 2A) or isolation site (carrier versus IPD; Fig. 2B). Phylogenetic analysis using the whole-genome sequence (Fig. S3) revealed a diverse genetic background for each 19A *cps* subtype (Fig. S3B and C), suggesting that the current distribution of 19A *cps* loci is partially a result of recombination events.

**TABLE 1:** S. pneumoniae isolates with a complete 19A *cps* locus included in this study

Origin	No. of isolates			
	Total	Pre-PCV (2004–2006)	Post-PCV7 (2006–2011)	Post-PCV10 (2011–2016)
Invasive pneumococcal disease	188	16	55	117
Carrier	148		115	33
Reference	2			

### The distribution of nonsynonymous SNPs across the capsular locus genes.

To identify potential functional consequences of the sequence variations, we identified all nonsynonymous single-nucleotide polymorphisms (nsSNPs) within the *cps* operon using the sequence from an IPD isolate from the Netherlands as a reference. This analysis initially revealed 49 unique *cps*-nsSNP types, for which most experiments were performed. Later, additional isolates were examined, resulting in 79 nsSNP types within 338 isolates. Table 2 shows to which previously described subtypes each SNP type belongs. Several mutation hot spots were observed, most notably within *rmlB* and *rmlD* (87.7 to 94.2% and 77.2 to 99.5% sequence similarity, respectively, compared to Hungary-19A-6), which are responsible for the synthesis of TDP-l-rhamnose, which is a component of the 19A CPS, and within the *wzg* gene (92.3 to 94.6%), a predicted regulator of CPS synthesis with the possible enzymatic function of linking the CPS to the cell wall peptidoglycan (26). Our analysis reveals that although these genes are mutation hot spots, the 19A *cps* subtypes (19A-I, 19A-II, 19A-II-Ins, 19A-III, 19A-IV, 19A-V, 19A-VI, and 19A-VIII) generally show a high similarity for the *rmlA* to *rmlD* gene cluster within each subtype, suggesting sequence conservation within the *cps* loci of the different 19A subtypes. Interestingly, the *rmlD* in subtypes 19A-I, 19A-II, 19A-II-Ins, and 19A-IV is situated in reverse direction and on the complement strand. Previously, Morona et al. reported a potential promoter upstream of *rmlD* on the opposite strand as that for *aliA*. They also described a stem-loop structure between *rmlB* and *rmlD* that could potentially function as a transcription terminator (22). Perhaps this reverse complement *rmlD* is regulated by another promoter. These subtypes also share the same *rmlB* and *rmlD* genes (100% similarity except for one point mutation in *rmlB* of SNP1). Subtypes 19A-III and 19A-V share a similar *rmlB* (99.90 to 100% similarity) and the same *rmlD* (100% similarity except for one point mutation for SNP30) genes. 19A-VI and 19A-VIII and the Hungary 19A-6 and Bentley 19A reference strain sequences comprise the other group containing similar *rmlB* and *rmlD* (99.4 to 100%) genes. The *wzg* sequences also show high similarity within the subtype, but fewer similarities are found between subtypes. Only subtypes 19A-III, 19A-V, and 19A-VI share the same *wzg* sequence (except one point mutation in SNP48). We observed that subtype V had an additional [YGX] repeat (4 *versus* 3 repeats) in the [YGX]_3_ repeat domain of the *wze* gene compared to the other subtypes. There is evidence that suggests that phosphorylation of tyrosine residues in that domain affects capsule levels (31).

**TABLE 2:** Distribution of the observed SNP types across the 19A subtypes^*a*^

19A subtype	SNP type(s)
I	SNP1–SNP17
II	SNP18–SNP23
II-ins	SNP24–SNP26
III	SNP30–SNP42
IV	SNP27–SNP29
V	SNP43–SNP45
VI	SNP46–SNP48
VIII	SNP49

a As described by Elberse et al. 2011 (23).

### Variations in the promoter region.

We also compared the promoter region of the *cps* operon based on the following four reference promoter elements described by Wen et al. (32): insertional element (IE), repeat unit of pneumococcus (RUP), spacing sequence (SS), and a core promoter sequence. All isolates had a promoter containing a largely conserved 31-nucleotide region prior to the start codon, followed by a completely conserved core promoter. Differences in the remainder of the promoter between the isolates can be largely grouped into two distinct promoter types. Type I starts with the IS*630* IE, followed by the RUP and the SS. Some strains in this group only contain a small partial IS*630* IE or do not have it at all. One isolate did not have a RUP, either, and had only a partial spacing sequence. In isolates of subtype 19A-IV, repeats of parts of the SS were found. Type II had its IE (IS*1201*) after its largely intact RUP, and only had a partial spacing sequence, showing the same differences observed by Wen et al. (32). The RUP in this group had an altered binding site, compared to the one described by Wu et al. (33), for transcription repressor CpsR, possibly affecting its binding. The distribution of the majority of the promoters, but not all, was linked to *cps* locus sequence and 19A *cps* subtype grouping (Fig. S3D). Two-thirds of subtype I had a type II promoter, and the remainder type I. Strikingly, SNP types SNP9 and SNP10 had identical CPS sequences, but either a type I or type II promoter. All of the other 19A *cps* subtypes had a type I promoter.

### The effect of nsSNPs on protein structure.

**FIG 3:**
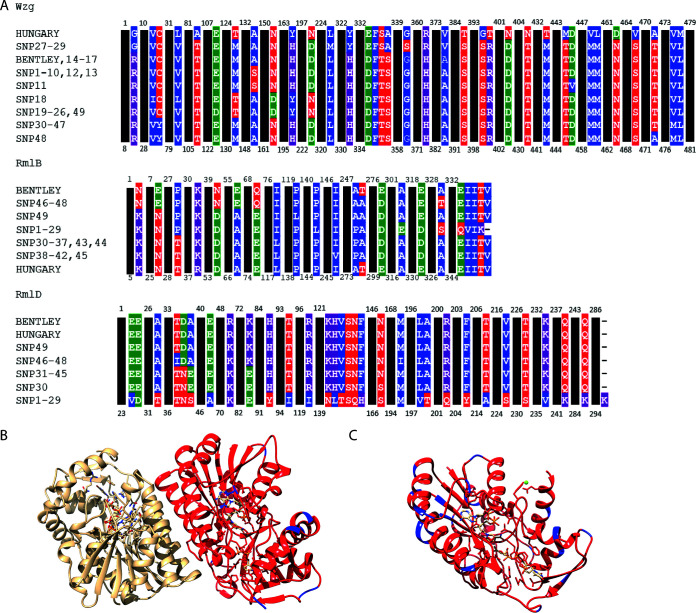
(A) Alignment of amino acid sequence from RmlB, RmlD, and Wzg of the different serotype 19A cpsSNP variants. Substitutions are colored as follows: blue, nonpolar amino acids; red, polar amino acids; green, negatively charged amino acids; purple, positively charged amino acids. All variants identified are shown. Structural model of an RmlB dimer (B) and of an RmlD monomeric protein (C). Differences in protein residues in the context of the RmlB and RmlD structures are shown in blue for the Hungary19A-6 reference protein sequence versus SNP2. The dimeric RmlB model is shown with one monomer color coded according to the sequence conservation and the other in a lighter color.

As the *rmlB* and *rmlD* genes contained the highest rates of nonsynonymous SNPs (nsSNPs) within the gene cluster, we decided to further investigate the protein sequence of RmlB and RmlD. We examined the amino acid sequence and structure of these enzymes of the TDP-rhamnose biosynthesis pathway (Fig. 3). Subtypes 19A-I, 19A-II, and 19A-IV all share identical RmlB and RmlD proteins, as do subtypes 19A-III and 19A-V (19A-VIII also shares the 19A-III and 19A-V RmlD). To determine the number of proteins that would be translated from the different alleles, the nucleotide sequence was converted to an amino acid sequence. For Wzg, RmlB, and RmlD, 9, 7, and 6 different proteins were found, respectively.

Next, we examined whether the nonsynonymous SNPs affected protein structure and function. Using protein structure analysis, we investigated the location of the mutations and their potential role in catalytic activity. The streptococcal RmlB proteins form a functional homodimer (34), while RmlD enzymes are monomeric (35). We systematically investigated the location of the 12 amino acid substitutions (compared to the Hungary reference sequence) that are the result of the SNPs in a structural model of RmlB, using the Streptococcus suis RmlB (90 to 92% sequence identity) structure as a reference (36). All residues are located on the surface of the RmlB homodimer, and none except K38 are in proximity to the substrate or cofactor binding site (Fig. 3B). A K38R mutations is found in the Hungary strain, with its side chain sitting on top of the aromatic ring of the nucleotide (Fig. 3B). However, the K-to-R mutation is a conserved mutation and should not impact the enzyme’s ability to bind the substrate. Importantly, nonsynonymous SNPs in RmlB were also not found at the dimer interface, which would potentially disrupt functionality. Therefore, the mutations appear to be subtle and are not likely to affect the RmlB protein structure, protein activity, and dimerization.

Interestingly, a higher rate of SNPs was found in RmlD (3 per 100 residues). RmlD is a monomeric protein in Gram-positive bacteria, and the structure of the closely related RmlD from Streptococcus pyogenes (78 to 81% sequence identity) was reported recently (35). A structural model of S. pneumoniae RmlD was built and analyzed to determine the potential impact of the nsSNPs (Fig. 3C). Like the nsSNPs found in RmlB, all nonsilent mutations were located on the surface of RmlD, facing away from the catalytic machinery.

### The consequence of nsSNPs in *rmlB* and *rmlD* on nucleotide sugar levels.

**FIG 4:**
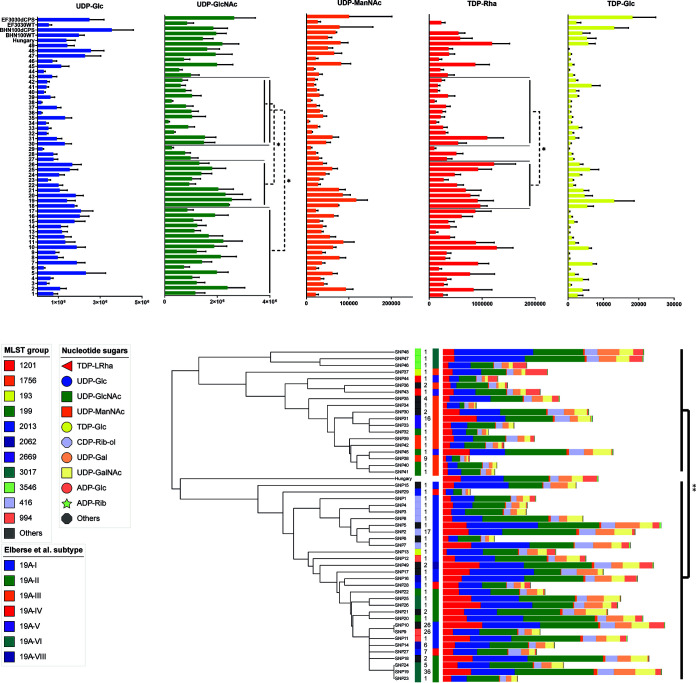
Results of mass spectrometry quantification of whole-cell monosaccharide and nucleotide sugar content in samples of 19A serotype isolates with a unique cpsSNP type. (Top) Quantities of the individual CPS monosaccharides or nucleotide sugars with standard error (SE) bars. The median differed significantly for all comparisons, as follows: UDP-Glc and UDP-GlcNAc (*P* < 0.01); UDP-ManNAc, TDP-Rha, and TDP-Glc (*P* < 0.05). Significant differences between subtypes I versus III (UDP-GlcNAc only), and II versus III (UDP-GlcNAc and TDP-Rha) are shown in the graph (*, *P* < 0.05) (Kruskal-Wallis). (Bottom) Phylogenetic tree of a *cps* locus MLST analysis with quantities of multiple monosaccharides and nucleotide sugars. Left to right: phylogenetic tree, genomic background (MLST group), prevalence in cohort (*n*), 19A *cps* locus subtype, and levels of monosaccharides. The following two phylogenetic groups can be distinguished: SNP1 to SNP29 plus SNP49 and SNP30 to SNP48. A significant difference between the two groups in the total amount of sugars is shown (**, *P* < 0.01). Also, GDP-Glc (*P* < 0.05) and CMP-Neu5Ac, UDP-Gal, UDP-Glc, UDP-GlcNAc, UDP-GalNAc, ManNAc, GDP-Fuc, and TDP-Rha (*P* < 0.01) differed significantly (multiple Mann-Whitney U tests). Data shown represent the results of three independent experiments.

To determine the consequence of nsSNPs in the *rml* genes we conducted sensitive ion pair ultrahigh-performance liquid chromatography–mass spectrometry (UHPLC-MS) to measure whole-cell levels of nucleotide sugars (37). Using the then-available 49 isolates, each with a unique cpsSNP type (SNP1 to SNP49), we focused on the building blocks of the 19A polysaccharide, namely, TDP-Rha, UDP-Glc, UDP-GlcNAc, UDP-ManNAc, and TDP-Glc (see Fig. 1 for the CPS synthesis by the CPS gene products). Levels of nucleotide sugars not related to the CPS were also analyzed to decrease the effect of changes in levels on the relative amounts measured. As a control, we used serotype 19F isolates EF3030 and BHN100 and their isogenic capsular knockout derivatives (38). Large variation in the amounts of nucleotide sugars produced by the 49 isolates was observed (Fig. 4). Strikingly, no clear correlation between 19A *cps* subtype or RmlB/RmlD/Wzg protein type (data not shown) and TDP-Rha production was observed. The EF3030 and BHN100 capsular knockouts showed no production of TDP-Rha, as expected. Interestingly, the unencapsulated strains seemed to have more of the other nucleotide sugars. When we compared nucleotide sugars as fractions of the total amount of measured sugar, we did not see distinct differences in the relative ratios of the nucleotide sugars (Fig. S5).

A significant difference in the median of the following nucleotide sugars was found: UDP-Glc, UDP-GlcNAc, UDP-Gal, UDP-GalNAc, and GDP-Man (*P* < 0.01) and UDP-ManNAc, TDP-Rha, TDP-Glc, and UDP-GlcA (*P* < 0.05), as well as the total amount of nucleotide sugars (*P* < 0.005) (Kruskal-Wallis). Significant differences between specific subtypes were only found recurring in subtype I versus III (UDP-GlcNAc and UDP-Gal) and II versus III (UDP-GlcNAc, UDP-Gal, UDP-GalNAc, TDP-Rha, and total sugar). No significant difference between subtypes was observed for the remainder of the nucleotide sugars.

The phylogenetic tree based on cpsMLST shows that the cpsSNP types are divided into two groups: SNP1 to SNP29 plus SNP49 and SNP30 to SNP48 (Fig. 4). Multiple Mann-Whitney U tests show that the former group produces significantly more monosaccharides in total (*P* < 0.01) and specifically for the following sugars: GDP-Glc (*P* < 0.05), CMP-Neu5Ac, UDP-Gal, UDP-Glc, UDP-GlcNAc, UDP-GalNAc, ManNAc, GDP-Fuc, and TDP-Rha (*P* < 0.01).

### Genetic variation of *wzg* and effect on capsule shedding.

**FIG 5:**
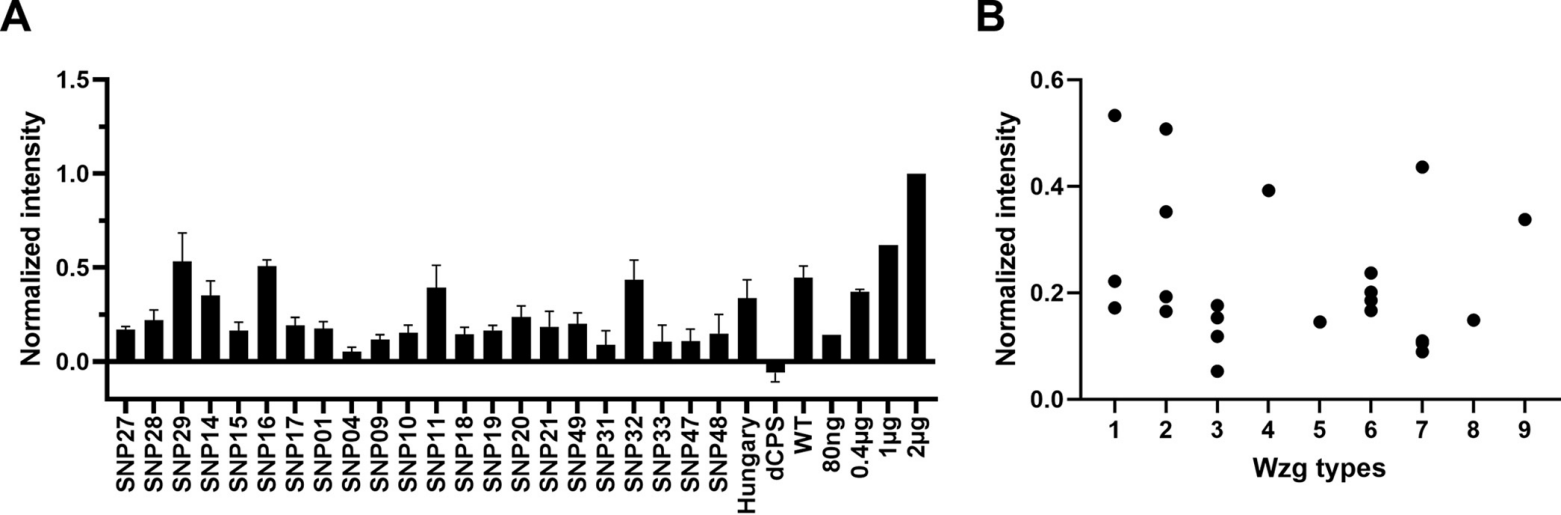
Western blot analysis of the effect of the Wzg sequence on capsule shedding. Supernatants of growth cultures blotted on a membrane were stained using serum from mice vaccinated with PCV13 (which includes serotype 19A). Shown intensities were corrected with a medium blank and normalized per blot using purified 19A CPS. (A) Mean intensity and SE of the different examined SNP types. Results of multiple independent experiments (*n* = 3) are shown. (B) Scatter blot of the different SNP types showing intensity variation within the Wzg protein type. No significant differences were found (Kruskal-Wallis).

The role of Wzg has yet to be elucidated, but it is thought to regulate expression of the *cps* operon; however, the mechanism remains unknown. Previously, others have described a potential enzymatic function for Wzg and other related proteins in B. subtilis, which suggests that Wzg is involved in coupling teichoic acids and capsular polysaccharides to the peptidoglycan layer (25, 26, 39). We hypothesized that the Wzg proteins (Table 3) translated from the distinct alleles, as observed within this cohort, affected the enzymatic function, which also might affect polysaccharide capsule shedding. To determine whether there was a difference between strains with the different *wzg* alleles in the amount of CPS shed, supernatants of isolate cultures were collected and transferred in triplicates to a membrane for Western dot blotting (Fig. S4), following a method similar to one previously described by Kietzmann et al. (27). Due to substantial proteinaceous background binding observed for anti-serotype 19 serum, we used serum from PCV13-vaccinated mice. Where possible, three or four isolates per Wzg protein type were examined. Although differences in shedding were observed (Fig. 5A), they could not be linked to amino acid sequence variation of Wzg proteins (Fig. 5B). Grouping of Wzg types based on >5 amino acid substitutions also could not be associated with specific shedding levels (data not shown), which suggests that there is an insignificant role in capsule shedding for the observed allelic variation in Wzg.

**TABLE 3:** Distribution of the observed SNP types across the Wzg types

Wzg type	SNP type(s)
1	SNP27–SNP29
2	SNP14–SNP17
3	SNP1–SNP10, SNP12, SNP13
4	SNP11
5	SNP18
6	SNP19–SNP26, SNP49
7	SNP30–SNP47
8	SNP48
9	Hungary

## DISCUSSION

After implementation of PCV7, changes in 19A subtype prevalence were observed (11), suggesting that the vaccination resulted in selective pressure, despite the fact that 19F and not 19A polysaccharides were included in this vaccine. We wished to further examine the distribution of the 19A subtypes and to find out what caused the change in prevalence. We discovered that the current prevalence resembled the prevalence of the early post-PCV7 vaccination period. Possibly, the initial change was due to an unfortunate coincidence of a low circulation of 19A, due to natural fluctuation, immediately after PCV7 introduction (40).

Using cpsMLST analysis, we found similar clustering of subtypes to that observed using PCR, but with greater resolution, showing variation within the subtype. As subtype distribution could not be related to either vaccination or disease, we could not identify the factors that drive the genetic diversity. The protein models of RmlB and RmlD, two of the proteins that show the most variation, showed no effect on either dimerization (RmlB only), substrate binding, or catalytic activity. Supporting this, TDP-Rha differences could not be linked to specific subtypes or Rml sequences, although a difference in the median of all subtypes was found. Also, it is possible that CPS thickness could be affected, even with similar TDP-Rha levels. However, we could not find, nor were able to design, a reliable method for measuring its thickness for such a large number of isolates. The resolution of documented methods, such as fluorescein isothiocyanate (FITC)-dextran exclusion (28) was too low to measure subtle differences in capsule thickness. It could be expected that Wzy, which is responsible for CPS polymerization, would be affected, if immunological pressure is selecting for mutations resulting in reduced or increased thickness of the capsule. However, this gene appeared to be highly conserved; hardly any SNPs were found in *wzy*. TDP-Rha was shown to inhibit the RmlA enzyme in a negative feedback loop, therefore regulating the amount of TDP-Rha being produced in bacteria (41). Therefore, it is also possible that the increased activity of RmlB and RmlD due to gain-of-function mutations (SNPs) is downregulated by the product inhibition of RmlA, leading to minimal differences in TDP-Rha synthesis as net result. Thus, the observed differences in TDP-Rha could be caused by a multitude of processes, such as an affected synthesis of TDP-Rha or its building blocks, other unknown factors regulating RmlA-D activity, and the thickness or shedding of the CPS. Capsular production could be enhanced, requiring more nucleotide sugars to be synthesized, resulting in the higher levels observed. Problems in the process of capsule formation could also possibly lead to a buildup of nucleotide sugars in which the lack of/or inhibition of certain enzymes could form a bottleneck, although buildup of specific monosaccharides would be expected there. Perhaps the observed difference in UDP-Glc and UDP-GlcNAc levels are a result of an impaired capsule formation. In this case, dTDP-Rha levels might not be different because of its negative feedback loop, which may also be occurring for UDP-ManNAc, although no evidence of this exists. The possibility also cannot be excluded that differences in specific and/or total monosaccharide/nucleotide sugar levels could also be attributed to genomic differences in genes outside the capsular locus.

We found large differences in whole-cell total sugar levels between the different serotype 19A strains grown under the same conditions. These surprising differences could point to major differences in sugar metabolism within this serotype, although differences in relative fractions of the individual sugars might then be expected. Although it is possible that the cultures grown to the same optical density differed in actual numbers of bacteria, it is unlikely that subtle variations in the actual bacterial load would lead to such significant differences. Possibly, the differences in total amount reflect differences in capsule thickness, where bacteria with thicker capsules have a higher total sugar count. A larger fraction of capsular sugars would be expected here, which was not what we observed. Possibly, upregulation of total sugar metabolism could be required for thicker capsules. Another possibility to explain the observed difference is different levels of shedding. Cells that shed more capsule may require a higher sugar metabolism. Alternately, smaller amounts of total sugars could be the result of higher levels of shedding, where shed capsule is lost in the sample preparation. However, we did not find a correlation between shedding levels and total sugar levels in the examined SNP types. The significant difference in total sugars between the two phylogenetically branched groups suggests that the *cps* locus is involved in this observed difference, although it remains unclear in what way. It will be interesting to find out if these differences in total amount of sugars translate to differences in other bacterial processes.

There could be multiple explanations for the existence of the variation in the *rmlB* and *rmlD* genes. Apparently, these genes allow accumulation of nsSNPs without affecting protein function, while this is not the case for *rmlA* and *rmlC*. Those genes might be more vulnerable for loss of function. In Pseudomonas aeruginosa, RmlA is a functional tetramer, with multiple multimerization domains and an allosteric pocket, in which mutations also cause inactivation of the enzyme (42). RmlB and RmlC both form a homodimer, but in RmlC, active site residues from both monomers are required (24, 34, 43). RmlD is monomeric and therefore potentially could have the most SNPs not affecting its function (35). Following this explanation, one could expect more random variation within these genes. However, variation seems to be conserved, resulting in a limited number of allele and protein variants. These alleles are largely distributed over two distinct groups, SNP1 to SNP29 plus SNP49 and SNP30 to SNP48. A difference in nucleotide sugar levels was also observed for these groups. Potentially, this is a result of a selective pressure, in the absence of PCV-13 as a selective pressure. Variation could have been caused by selection for metabolic advantages in certain environments. Another possibility is that we are simply observing the natural genetic drift of the 19A serotype. The observed difference could also be the result of multiple imperfect recombination events—the 19A *cps* locus is found in different genomic backgrounds, and the most genetically diverse genes are on the edges of the *cps* locus. More experiments are required to further understand this distribution and the possible selective pressure behind it.

Wzg also showed a high level of SNPs. As it is shown to be involved in CPS synthesis and is required for normal CPS levels (26), it is possible that differences in sequence could affect capsule thickness. Bacteria lacking *wzg* have a decreased amount of capsule (26). A decrease in CPS expression might also lead to a decrease in shedding (28). The different levels of sugars observed could therefore also be an indicator for differences in shedding. In our experiments, we observed large differences in capsule shedding; however, this was not associated with *wzg* sequence variation.

We also reported differences in the *cps* locus promoter, even within 19A subtypes. We have yet to pursue further examination of their relevance. It will be interesting to determine which consequences these differences have on gene expression and whether they confer an evolutionary advantage. Possibly invasiveness, disease progression, or colonization are affected.

To conclude, we have shown, with increased resolution, the variation within the 19A serotype in a cohort from the Netherlands, observing 79 nonsynonymous cpsSNP types. We were unable to find a correlation between cpsSNP type and isolate origin. However, we did observe conserved *wzg*, *rmlB*, and *rmlD* alleles within the previously described 19A subtypes. Further studies are required to investigate the drivers of the genetic variation in the *cps* locus described in this study. This will lead to improved understanding of the effects of host-pathogen interactions that result in pneumococcal capsule variation and its dynamics.

## MATERIALS AND METHODS

### Genetic analysis of S. pneumoniae isolates.

The cohort consisted of patients from all age groups diagnosed with invasive pneumococcal disease admitted to 22 Dutch hospitals (having blood cultures assessed in 9 sentinel laboratories) between 2004 and 2016 (30). Blood isolates were obtained from the Netherlands Reference Laboratory of Bacterial Meningitis (NRLBM) and the Pneumococcal Bacteraemia Collection Nijmegen (PBCN). Additional isolates from a collection of S. pneumoniae carriers were obtained from the National Institute for Public Health and the Environment (RIVM), which was collected for screening serotype prevalence spanning the period of 2006 to 2016. The isolates were subjected to whole-genome sequencing, which was performed with BaseClear using Illumina next-generation sequencing (NGS). CPS loci from Elberse et al. (23) were obtained by Sanger sequencing. Assemblies were constructed in CLC Genomics Workbench. Genome assembly of short reads into contigs was performed using SPAdes; contigs were subsequently assigned a taxonomic origin with Kraken2. All DNA sequences identified as pneumococcus were used for further analysis. The NGS data were used for cpsMLST and whole-genome MLST (wgMLST) analyses using SeqSphere software version 6.0.2 (Ridom GmbH, Münster, Germany). The cpsMLST scheme was based on all genes in the 19A *cps* operon, and the in-house wgMLST scheme comprised 1,942 genes (1,210 core genome and 732 accessory genome targets) using S. pneumoniae TIGR4 (GenBank accession number NC_003028.3) as a reference genome. Bionumerics was used for MLST minimum spanning tree analysis, for sequence alignment, and for calling of nonsynonymous single nucleotide polymorphisms. 19A reference strains were obtained from Bentley et al. (9) and McGee et al. (Hungary 19A-6) (44).

### Mass spectrometry analysis of nucleotide sugars.

The Streptococcus pneumoniae 19A strains were grown overnight on blood agar plates (Columbia III agar with 5% sheep blood, catalog no. 254098, containing 12 g/liter pancreatic digest of casein, 5 g/liter peptic digest of animal tissue, 3 g/liter yeast extract, 3 g/liter beef extract, 1 g/liter corn starch, 5 g/liter sodium chloride, 13.5 g/liter agar, 4 g/liter growth factors, and 5% sheep blood [defibrinated] [pH 7.3 ± 0.2]; BD) at 37°C and 5% CO_2_. The next day, single colonies were inoculated into 30 ml Todd-Hewitt broth supplemented with yeast extract (THY) broth (catalog no. 249240 [Difco] and SA-NV CON.1702 [Labconsult]; 3.1 g/liter heart, infusion from 500 g, 20 g/liter Neopeptone, 2 g/liter dextrose, 2 g/liter sodium chloride, 0.4 g/liter disodium phosphate, 2.5 g/liter sodium carbonate, and 5 g/liter yeast) in 50-ml tubes at 37°C and 5% CO_2_ and grown until an optical density measured at a wavelength of 620 nm reached 0.3. Subsequently, samples were immediately placed on ice with NaCl (catalog no. 1064041000; Merck) and spun down by centrifugation (1 min, precooled centrifuge at 4°C and 3,220 × *g*). The supernatant was discarded and the pellet was washed with 1.8 ml wash buffer (75 mM ammonium carbonate, catalog no. 207861; Sigma) in Milli-Q water, buffered with acetic acid (pH 7.4, catalog no. A6283; Sigma) and cooled to 4°C prior to use. The final suspension was transferred to a 2-ml tube and spun down by centrifugation (1 min at 4°C and 25,000 × *g*). The supernatant was discarded and the pellet was stored at −80°C until sample preparation. Frozen cells were extracted at −20°C with 1 ml precooled 2:2:1 (vol/vol/vol) methanol-acetonitrile-water (methanol, catalog no. 20846.361 [VMR]; acetonitrile, catalog no. 1000291000 [Merck]; water, catalog no. 3624331 [B. Braun Medical]) for 5 min. Subsequently, this was centrifuged at 25,000 × *g* for 3 min at 4°C. The resulting supernatants were dried overnight using a vacuum centrifuge at room temperature, and the pellets were stored at−80°C.

UHPLC–multiple-reaction monitoring (MRM) acquisition and data analysis were performed as previously described (37).

### Protein structure analysis.

The structural model for the RmlB dimer and the RmlD monomer based on the S. pneumoniae Hungary19A-6 subtype were built using the SWISS-MODEL server (45). Structural templates were 1ker.pdb for RmlB (36) and 4wpg.pdb and 1kc3.pdb for RmlD (35, 46). Molecular graphics and analyses were performed with the UCSF Chimera package, developed by the Resource for Biocomputing, Visualization, and Informatics at the University of California, San Francisco (supported by NIGMS P41-GM103311) (47).

### Capsule shedding analysis.

S. pneumoniae 19A isolates were grown overnight as previously described. The next day, bacteria were harvested from the plates and inoculated into 45% M17-45% CAT-10% fetal calf serum (FCS) broth with catalase (16.3 g/liter M17, 4.5 g/liter Casamino Acids, 2.25 g/liter sodium chloride, 2.25 g/liter Bacto tryptone, 4.5 g/liter yeast extract, 2.25 g/liter glucose, and 22 U/ml catalase [catalog no. C3155; Sigma-Aldrich]) in 10-ml tubes at 37°C and 5% CO_2_ and grown until an optical density measured at a wavelength of 620 nm reached 0.3 to 0.5 (starting optical density, 0.05). Samples were then spun down by centrifugation (10 min, room temperature, 3,220 × *g*), and supernatant was collected. Then, remaining bacteria were removed using 0.2 μm polyethersulfone (PES) membrane syringe filters. Then, the supernatant was diluted in phosphate-buffered saline (PBS) (1:5) before application to a nitrocellulose membrane using an acrylic Minifold I dot blot 96-well plate system (catalog no. 10447900; GE Whatman). Afterwards, the membrane was blocked using 5% bovine serum albumin (BSA) in PBS for 1 h at room temperature while shaking. Then, the membrane was incubated in 0.5% BSA, 0.1% Tween, 0.1 to 0.2% serum from PCV13-vaccinated mice (containing 22.29 μg/ml anti-19A IgG) in PBS for 1 h at room temperature while shaking. Membranes were then washed 5× for 5 min each in PBS with Tween 20 (PBST) and incubated in 0.5% BSA, 0.1% Tween, 0.01% rabbit-horseradish peroxidase (HRP) anti-mouse serum in PBS for 1 h at room temperature while shaking. After another round of washing (5 × 5 min in PBST), ECL Plus reagent (GE) was applied, and luminescence was detected using a ChemiDoc XRS+ system (Bio-Rad). Intensity was corrected to growth culture optical density to mitigate the influence of the difference in number of bacteria per sample.
