# Supplementary material for: Examining the Distribution and Impact of Single-Nucleotide Polymorphisms in the Capsular Locus of Streptococcus pneumoniae Serotype 19A
Source: Infect Immun. 2021 Oct 15;89(11):e00246-21. doi: 10.1128/IAI.00246-21 (PMC8519296; doi:10.1128/IAI.00246-21)
Supplement: Supplemental file 1 — Supplemental material. Download IAI.00246-21-s0001.pdf, PDF file, 0.4 MB [file iai.00246-21-s0001.pdf]

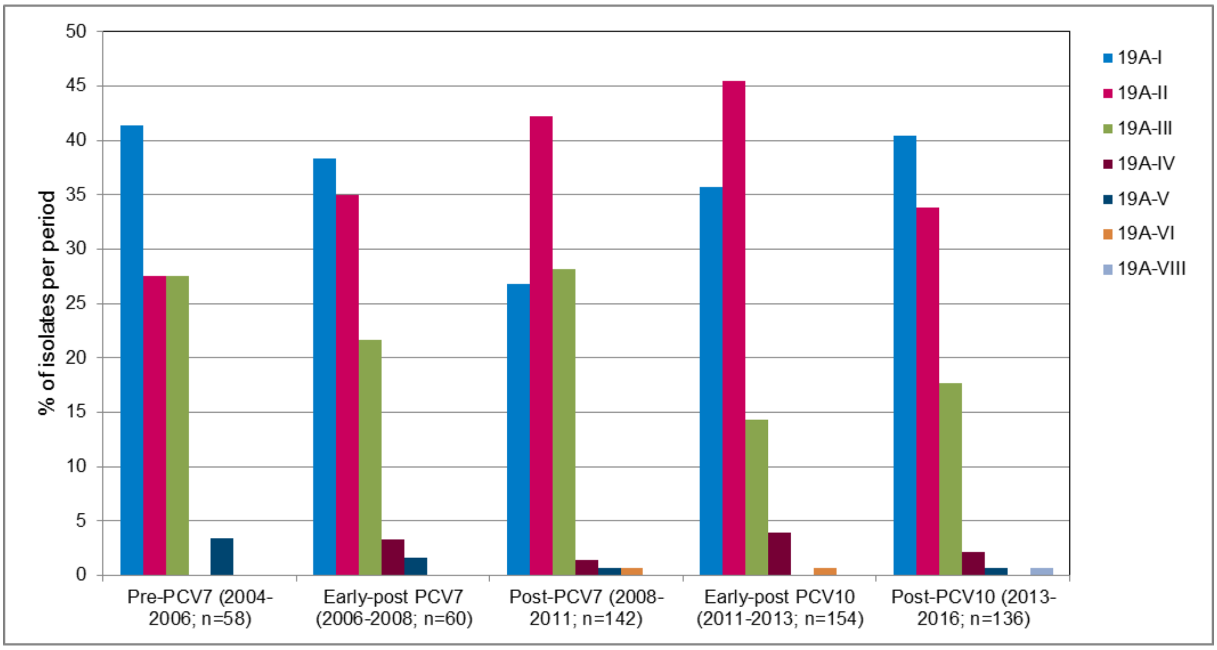

Supplementary Figure 1: Distribution of 19A-subtypes in IPD isolates from the Netherlands. Subtypes are assigned based on the previously described allele-specific PCR screening method (Elberse *et al.* 2011). Time periods at which isolates were collected are indicated below the X-axis. Data is represented as percentage of the subtype within the population examined in the indicated time period.

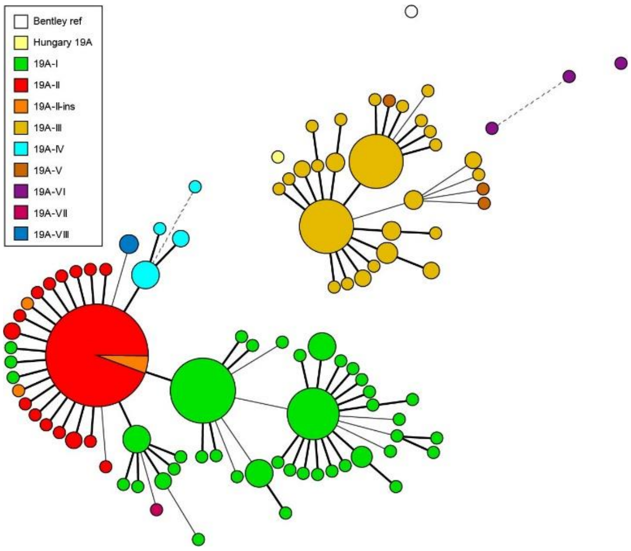

Supplementary figure 2: Multiple spanning tree based on the cpsMLST analysis of our cohort. The distribution of the different 19A-subtypes (colors) as described by Elberse *et al.* 2011.

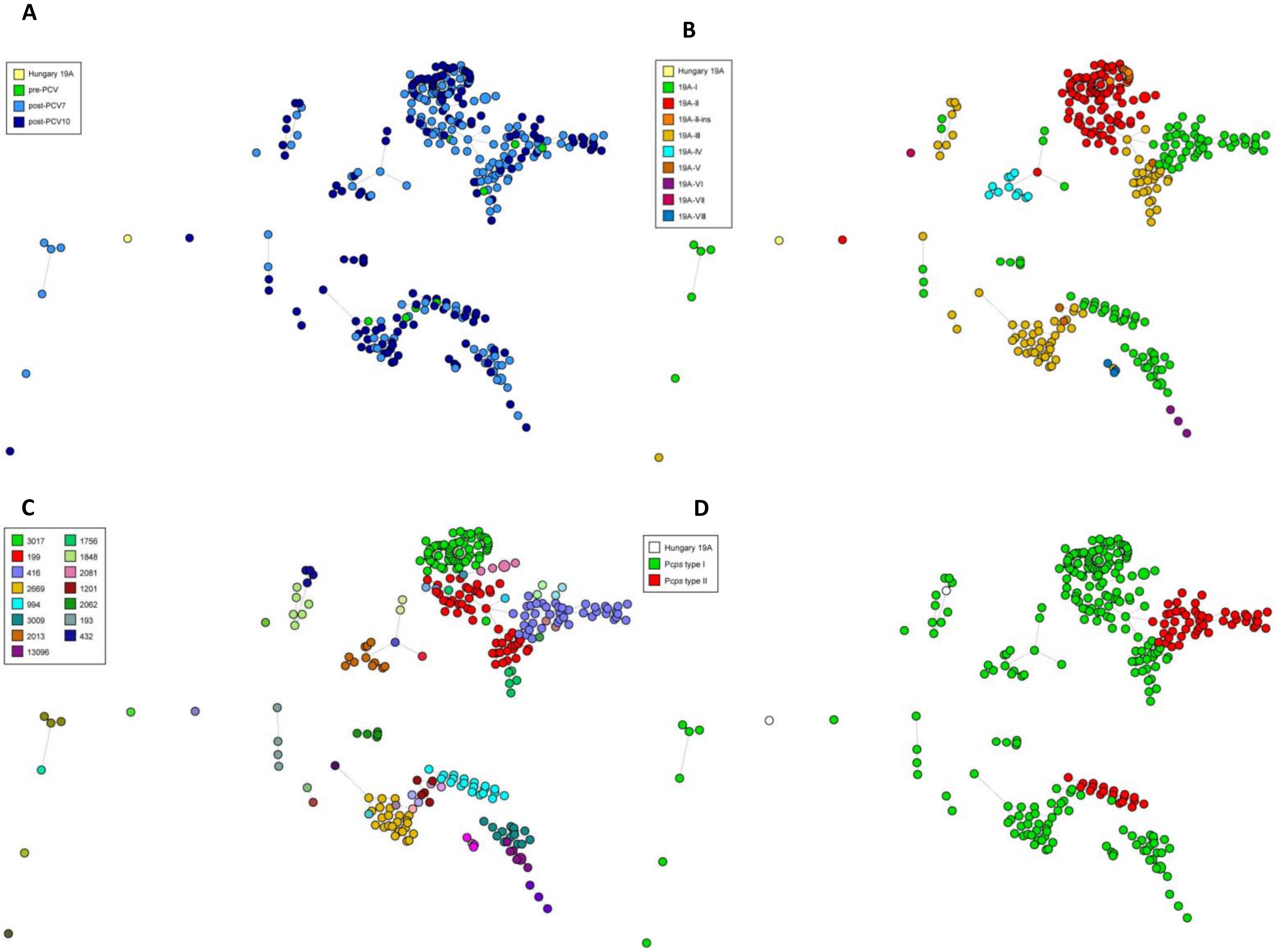

Supplementary figure 3: Multiple spanning tree of an MLST analysis based on the whole genome. Distribution of pre and post-vaccination isolates (A), serotype 19A-subtypes as described by Elberse *et al.* 2011 (B), MLST-groups (C), and CPS locus promoter types (D) in our cohort.

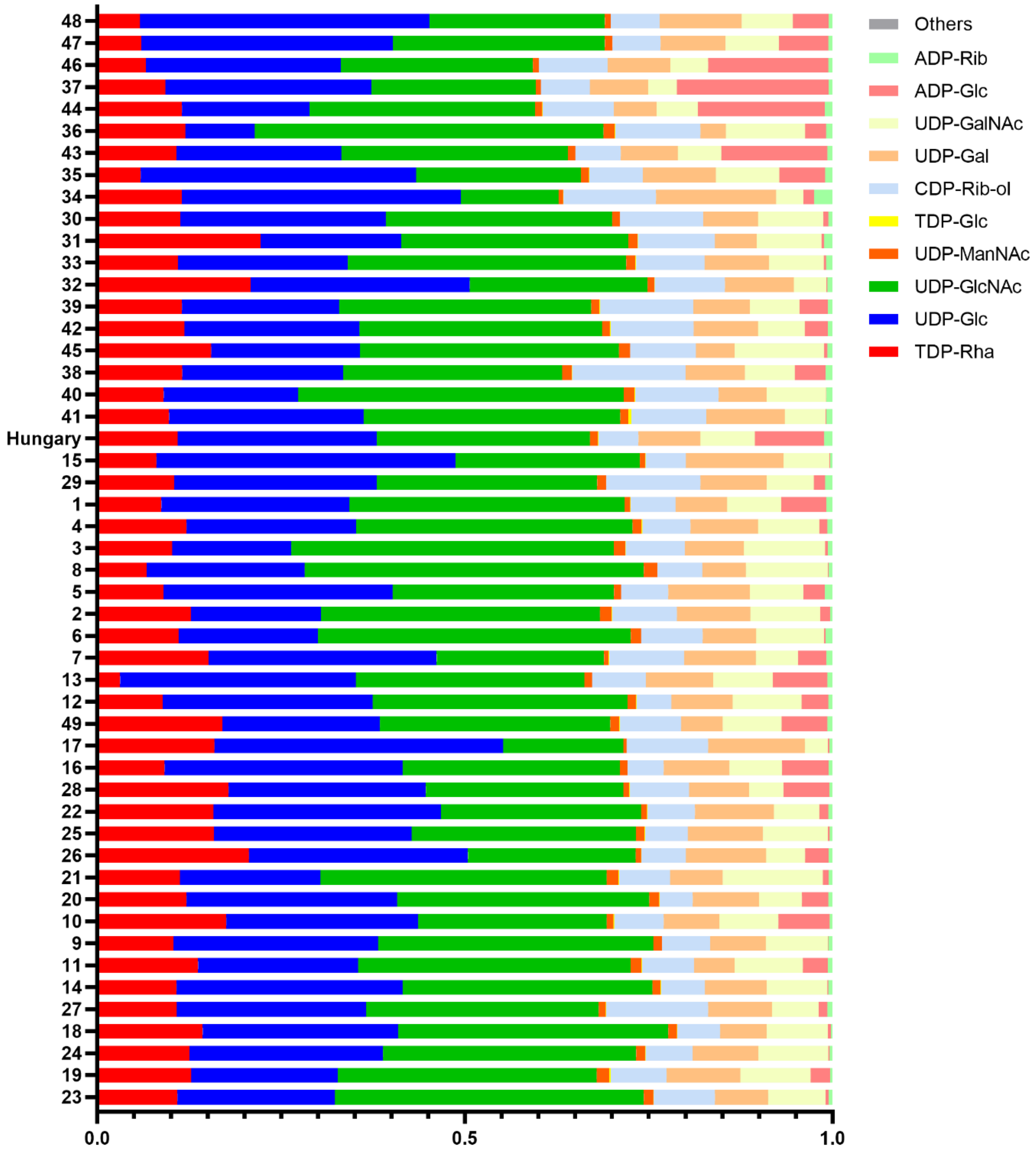

Supplementary figure 4: Relative levels of monosaccharides and nucleotide sugars. Mass spectrometry results of each sugar were normalized to the total amount of sugars measured for each SNP type. The order of SNP types is the same as in the phylogenetic tree of Figure 4.

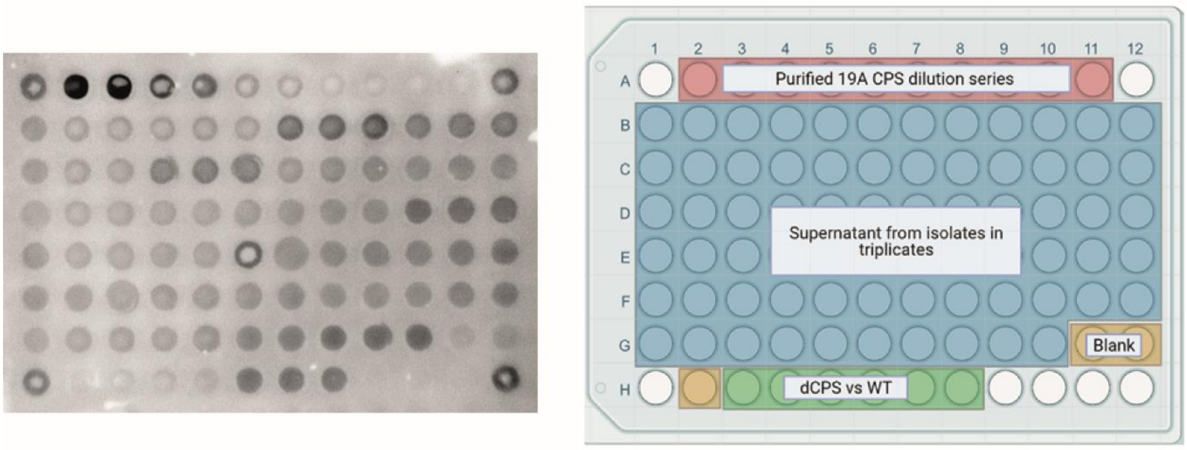

Supplementary figure 5: (Left) Example of a Western dot-blot using anti-CPS serum on supernatant from growth culture to assess levels of shedding. (Right) Schematic representation showing which samples were loaded.
